# Alginic Acid-Coated Chitosan Nanoparticles Loaded with Legumain DNA Vaccine: Effect against Breast Cancer in Mice

**DOI:** 10.1371/journal.pone.0060190

**Published:** 2013-04-05

**Authors:** Ze Liu, Dan Lv, Shu Liu, Junbo Gong, Da Wang, Min Xiong, Xiaoniao Chen, Rong Xiang, Xiaoyue Tan

**Affiliations:** 1 Medical School of Nankai University, Tianjin, China; 2 The Moores Cancer Center, University of California San Diego, La Jolla, California, United States of America; 3 Tianjin Key Laboratory of Modern Drug Delivery and High Efficiency in Tianjin University, Tianjin, China; 4 Key Laboratory of Functional Polymer Materials of Ministry of Education in Nankai University, Tianjin, China; The Ohio State University, United States of America

## Abstract

Legumain-based DNA vaccines have potential to protect against breast cancer. However, the lack of a safe and efficient oral delivery system restricts its clinical application. Here, we constructed alginic acid-coated chitosan nanoparticles (A.C.NPs) as an oral delivery carrier for a legumain DNA vaccine. First, we tested its characteristic in acidic environments *in vitro*. DNA agarose electrophoresis data show that A.C.NPs protected DNA better from degradation in acidic solution (pH 1.5) than did chitosan nanoparticles (C.NPs). Furthermore, size distribution analysis showed that A.C.NPs tended to aggregate and form micrometer scale complexes in pH<2.7, while dispersing into nanoparticles with an increase in pH. Mice were intragastrically administrated A.C.NPs carrying EGFP plasmids and EGFP expression was detected in the intestinal Peyer’s patches. Full-length legumain plasmids were loaded into different delivery carriers, including C.NPs, attenuated *Salmonella typhimurium* and A.C.NPs. A.C.NPs loaded with empty plasmids served as a control. Oral vaccination was performed in the murine orthotopic 4T1 breast cancer model. Our data indicate that tumor volume was significantly smaller in groups using A.C.NPs or attenuated *Salmonella typhimurium* as carriers. Furthermore, splenocytes co-cultured them with 4T1 cells pre-stimulated with CoCl_2_, which influenced the translocation of legumain from cytoplasm to plasma membrane, showed a 4.7 and 2.3 folds increase in active cytotoxic T lymphocytes (CD3^+^/CD8^+^/CD25^+^) when treated with A.C.NPs carriers compared with PBS C.NPs. Our study suggests that C.NPs coated with alginic acid may be a safe and efficient tool for oral delivery of a DNA vaccine. Moreover, a legumain DNA vaccine delivered orally with A.C.NPs can effectively improve autoimmune response and protect against breast cancer in mice.

## Introduction

DNA-based vaccines provide protection against cancers in a variety of animal models [1], [2], [3], [4]. Upon vaccination, host autoimmunity is activated, resulting in significant suppression of tumor growth and metastasis [5], [6], [7], [8], [9]. Although conventional cancer DNA vaccines are designed to target tumor cells, more novel vaccines are being developed to target the specific contents in the tumor microenvironment [1], [6], [10], [11]. Legumain, an asparaginyl endopeptidase, is significantly overexpressed on tumor-associated macrophages [6], [12]. Moreover, DNA vaccines targeting legumain and delivered by attenuated *Salmonella typhimurium* exhibited efficiency in improving both the survival time of tumor-bearing mice and reducing tumor growth [1], [13]. Given the promising results from previous animal experiments, it may be that a legumain DNA vaccine could be used to treat cancer in the humans. However, there are several issues preventing the clinical application of this potentially powerful therapeutic strategy. One significant obstacle is the lack of a suitable carrier [14].

Oral vaccination has advantages over intravenous administration, as it is noninvasive, more convenient, and achieves better clinical compliance [15]. In terms of biological carriers, both viral vectors and bacteria are used for oral DNA vaccination [16], [17]. Bacteria-based delivery systems have been shown to be more effective in priming immune responses, able to be loaded with larger amounts of DNA clones, and more easily controlled compared with viral vectors [13]. Nonetheless, safety remains an issue when applying them to the humans. Hence, studies have been carried out to develop novel delivery carriers with low toxicity and high efficiency. In the past decade, chitosan nanoparticles (C.NPs) have emerged as novel carrier candidate because of their excellent stability, capacity to enhance mucosa absorption, and good compatibility with vaccine DNA [18], [19]. As a natural biopolymer derived from crustacean shells, C.NPs possess ideal properties of polymeric carriers; they are biocompatible, biodegradable, non-toxic, and inexpensive [20], [21]. Studies show that C.NPs less than 500 nm in diameter are transported through the intestinal mucosa via an endocytotic mechanism [18], [19], [20]. Moreover, improved mucoadhesion and transient opening of tight junctions in the mucosal cell membrane contribute to the absorption-promoting effect of chitosan. By incorporating DNA plasmids into C.NP systems, oral DNA vaccines can be delivered to major targets, the Peyer’s patches, and be taken up by antigen-presenting cells [18], [20], [22], [23], [24].

Although C.NPs have numerous advantages as delivery carriers for oral DNA vaccination, DNA degradation in the gut and low uptake efficiency in the gastrointestinal lymphoid tissue largely hamper their development. Recently, a delivery system composed of a chitosan core coated with sodium alginate has been described [25]. Borges et al. demonstrated that sodium alginate-coated nanoparticles were readily taken up by rat Peyer’s patches. Release profiles showed that burst release of loaded ovalbumin in pH of 1.2 (simulated gastric fluid) was largely prevented by its entrapment in alginate-coated chitosan nanoparticles. This delivery system also acted as an effective adjuvant for hepatitis B surface antigen when subcutaneously administered in a mouse model [26].

Here, we hypothesized that alginic acid-coated chitosan nanoparticles (A.C.NPs) loaded with DNA plasmid could resist DNA degradation in the acidic gastric environment and be effectively taken up and expressed by antigen-presenting cells in the Peyer’s patches. In addition, we examined the therapeutic effect of a legumain DNA vaccine using this delivery system in a mouse breast cancer model.

## Materials and Methods

### Ethics Statement

The animal experiments were performed in accordance with institutional guidelines, and the study was approved by the ethics committee of Nankai University. Mice were anesthetized with a mixture of oxygen/isoflurane before each experiment to alleviate their suffering. For survival data, humane endpoints were chosen to terminate the distress of the experimental animal via carbone dioxide euthanasia. The animals were monitored twice per day and extreme anorexia (poor appetite and emaciated appearance) was determined as a humane endpoint.

### Polymers and Reagents

Chitosan (C-3646, MW: 810,000; degree of deacetylation 75–85%) [27] and alginic acid (A 7003, MW: 240,000) were purchased from Sigma-Aldrich (St. Louis, MO). All reagents used were of analytical grade and all solutions were prepared in MilliQ water. EGFP expression plasmids were constructed by ligating the EGFP coding region into the pcDNA3.1 expression vector. EGFP was cloned from pEGFP-N1 (Clontech, Mountain View, CA) and pcDNA3.1 (+) plasmids was purchased from Invitrogen (Carlsbad, CA). Legumain expression plasmids (pCMV-Legumain) were kindly provided by Dr. Ralph Reisfeld (The Scripps Research Institute, La Jolla, CA).

### Preparation of A.C.NPs Loaded with DNA Plasmid

**Figure 1:**
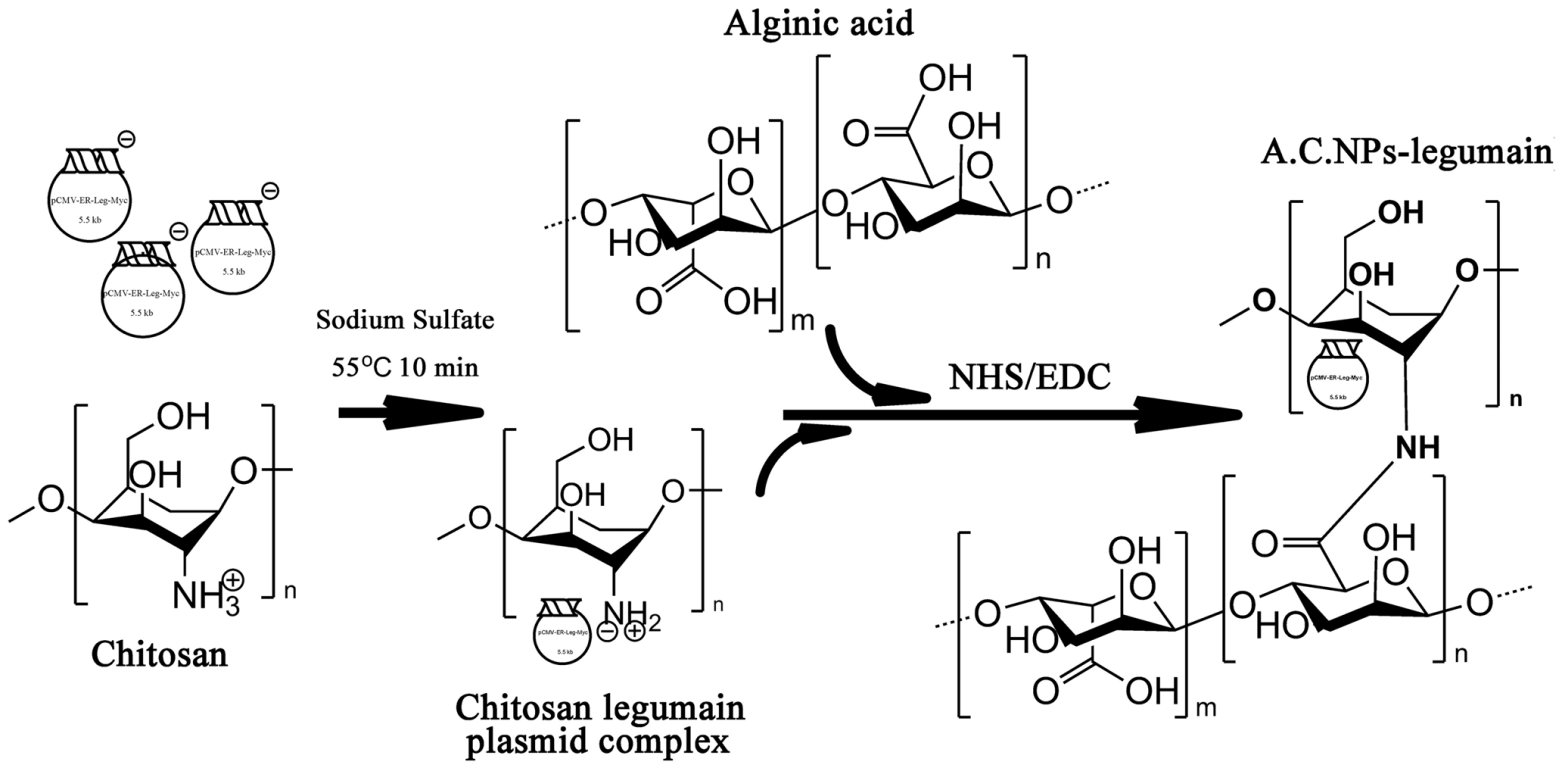
Illustration of A.C.NPs-legumain synthesis.

C.NPs loaded with EGFP or legumain DNA plasmid (C.NPs-EGFP or C.NPs-legumain) were prepared using a stirring method described as follows [28]: 0.2% (w/v) chitosan solution prepared in 25 mM sodium acetate buffer (pH 5.2) and 200 µg/mL DNA in 25 mM of sodium sulfate solution were preheated to 55°C separately. Equal volumes of both solutions were mixed together and vortexed for 10 minutes at room temperature. A.C.NPs loaded with DNA plasmids (A.C.NPs-EGFP or A.C.NPs-legumain) were prepared as follows. First, 1% (w/v) alginic acid solution in MilliQ water was preheated to 55°C. Equal volumes of alginic acid and C.NPs-EGFP (or solution of C.NPs-legumain) solutions were then mixed. The mixture was added to an EDC/NHS solution, and the pH adjusted to 9.0 for crosslinking, 2 h at room temperature. A.C.NPs-EGFP or A.C.NPs-legumain was then passed through a PD-10 desalting column (GE healthcare, Buckinghamshire, UK), and the NPs eluted by PBS to remove salts (Fig. 1). All nanoparticles not immediately used were stored at 4°C for no longer than 2 weeks. Sonication was required before nanoparticles were ready to use.

### Characteristics of A.C.NPs

Morphological examination of A.C.NPs was performed using Philips T20ST transmission electron microscopy (TEM, Amsterdam, Netherlands). Briefly, A.C.NPs-legumain and C.NPs-legumain were resuspended in MilliQ water (pH 7.0) or hydrochloric acid buffer (pH 1.5) without fixation or staining. Samples were then adhered to a carbon-coated copper grid and dried under atmospheric pressure at room temperature. The TEM acceleration voltage was 200 kV.

Nanoparticle size and zeta potentials were measured using a ZetaPALS/90 plus zeta potential analyzer (Brookhaven Instruments Corp, Holtsville, NY). Fourier transform infrared (FT/IR) spectroscopy was used to identify the chemical structure of the nanoparticles. The coated particles were washed with MilliQ water, centrifuged and the sediment freeze-dried overnight (Labconco, Kansas City, KS). The coated and uncoated particles were desiccated at room temperature until analysis. The IR spectra of the samples were recorded using a Tensor 27 FT-IR spectrophotometer (Bruker, Ettlingen, Germany). The spectra were the average of 50 scans recorded at a resolution of 2 cm^−1^ from 4000 to 40 cm^−1^.

### Agarose Gel Electrophoresis of DNA Loaded in A.C.NPs or C.NPs

Given the tendency of A.C.NPs to aggregate in low pH solutions, we further evaluated the protective effect of A.C.NPs against DNA degradation in acidic environments. A.C.NPs-legumain, C.NPs-legumain, and naked legumain DNA plasmids were treated with 31.6% (v/v) hydrochloric acid buffer (pH 1.5) for 0.5, 1, 2, or 4 h. Naked plasmid DNA dissolved in MilliQ water (pH 7.0) and empty A.C.NPs in hydrochloric acid buffer (pH 1.5) were used as positive and negative controls, respectively. Samples were evaluated by 1% (w/v) agarose gel electrophoresis at 120 V for 20 min (Bio-Rad Life Science, Hercules, CA) and the bands stained with ethidium bromide. Images were taken using a UV transilluminator (Bio-Rad).

### Animals and Cell Lines

Female BALB/c mice that weighed approximate 20 g were obtained from the Academy of Military Medical Sciences, Laboratory Animal Center (Peking, China). Mice were housed in the animal facilities of Nankai University with access to food and water. 4T1 murine breast carcinoma cells were obtained from the American Type Culture Collection (Manassas, VA) and cultured in RPMI-1640 medium supplemented with 10% fetal bovine serum (Invitrogen, Carlsbad, CA).

### Uptake and Expression in the Peyer’s Patches

Twelve female BALB/c mice were randomly subjected to oral administration of 200 µl EGFP plasmid (20 µg plasmid DNA), the equivalent amount of C.NPs-EGFP, or the equivalent amount of A.C.NPs-EGFP once a day for 3 days (n = 5). The animals were sacrificed 24 h after the last treatment and their intestinal Peyer’s patches were isolated. One section of the freshly isolated tissues were immediately frozen in O.C.T. compound and stored at −80°C for immunofluorescence staining while the remaining portions were subjected to physically grinding prior to flow cytometry analysis.

### Tumor Growth and Survival Time

Animals were randomly divided into 5 groups (n = 10): PBS control, C.NP-legumain, A.C.NPs-legumain, A.C.NPs, and *S Typhi*-legumain groups. On the first day of experiments, 5×10^4^ 4T1 cells suspended in 50 µl PBS buffer were injected orthotopically into the fourth mammary fat pad (left) of mice. From the fourth day on, mice were orally administrated 200 µl legumain DNA (20 µg plasmid DNA), delivered in different carriers or the vehicle control once a day for 5 days. Tumor dimensions were measured using calipers. Tumor volume was calculated using the formula, Tumor Volume = (a^2^×b)/2, where “a” and “b” are the two perpendicular diameters, with “a” being the larger one [29]. Body weight of each mouse was also recorded to calculate tumor weight/body weight ratios.

### Cytotoxic T-lymphocyte (CTL) Killing Assay

Mice were sacrificed when the primary tumors reached approximately 500 mm^3^ in volume (about 21 d after injection). Spleens were surgically removed and physically ground into dispersed single cells. Co-culture was performed by seeding the isolated splenocytes into cultures of adherent 4T1 cells, which were or were not previously stimulated for 24 h with CoCl_2_. After 24 h co-culture, splenocytes in the cultural medium were collected and analyzed via flow cytometry.

### Immunofluorescence Staining

Immunofluorescence staining was performed as previously described [6]. Briefly, O.C.T. embedded blocks were sliced into 5-µm-thick sections. Slides were then fixed in cold acetone, blocked in 10% goat serum, and stained with PE-labeled anti-F4/80 antibody (1∶100 dilution, eBioscience, San Diego, CA). Cell nuclei were stained using mounting medium with DAPI (Vector Laboratories, Inc. Burlingame, CA). Double staining for E-cadherin and legumain was performed on 4T1 cells. 4T1 cells cultured in 6-well plates were fixed in methanol and blocked with 10% goat serum. Cells were incubated with the following primary antibodies: anti-E-cadherin (1∶200 dilution, Cell Signaling Technology Inc., Danvers, MA) and rabbit anti-mouse legumain (1∶100 dilution, Santa Cruz, CA). After 24 h, the samples were incubated with fluorescein-conjugated secondary antibodies. Cell nuclei were visualized using mounting medium with DAPI. Photos were taken with TCS-SP5 fluorescence confocal microscopy (Leica Microsystems, Mannheim, Germany).

### Flow Cytometry Assay

To quantify uptake and expression of plasmids in the Peyer’s patches, dispersed cells were incubated with PE-labeled anti-F4/80 antibody (1∶100 dilution) and APC-labeled anti-CD11c (1∶200 dilution, BD Pharmingen, San Diego, CA), then analyzed with 4-color flow cytometry (BD, Franklin Lake, NJ). To perform the cytotoxic T lymphocyte (CTL) killing assay, splenocytes were stained after co-culture with PE-labeled rat anti-CD3 (1∶100 dilution), APC-labeled anti-CD4, anti-CD8a (1∶200 dilution) and FITC-labeled anti-CD25 (1∶200 dilution), and analyzed with flow cytometry. All fluorescein-conjugated antibodies were purchased from BD Pharmingen. Data were analyzed with FlowJo software (Tree Star, Inc, Ashland, OR).

### Statistical Analysis

Statistical analysis was done with GraphPad Software (La Jolla, CA). Data were expressed as mean ± SD of triplicate samples, from at least three independent experiments. Statistical significance was determined using Student’s t-test or two-way ANOVA.

## Results

### Characteristics of A.C.NPs/C.NPs Loaded with Legumain DNA

**Figure 2:**
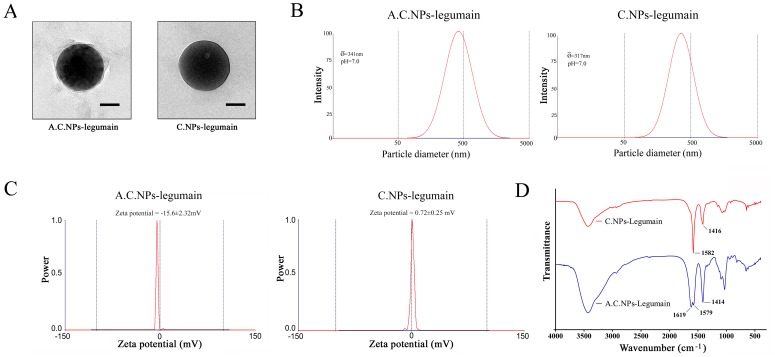
Characteristics of A.C.NPs-legumain and C.NPs-legumain. (A)Representative images of A.C.NPs- legumain (left) and C.NPs-legumain (right) observed by TEM. Scale bar = 100 nm. (B& C) Size distributions in purified water (pH7.0) and the mean value of zeta-potentials of A.C.NPs-legumain and C.NPs-legumain at 37°C. (D) FTIR spectra of uncoated chitosan particles and alginate acid-coated chitosan particles.

Morphology of A.C.NPs and C.NPs loaded with legumain DNA was analyzed by TEM. A.C.NPs-legumain were round, exhibiting a distinct alginic acid layer around the chitosan core when compared with the non-coated C.NPs-legumain (Fig. 2A). Size and zeta potential of the A.C.NPs-legumain were measured and compared with those of C.NPs-legumain at pH 7.0. As shown in Fig. 2B, C.NPs-legumain and A.C.NPs -legumain were 317±19.3 nm and 341±27.5 nm in diameter, respectively (Fig. 2B). The zeta potential of C.NPs-legumain was 0.72±0.25 mV while the zeta potential of A.C.NPs-legumain was −15.6±2.32 mV (Fig. 2C). The FTIR spectra of C.NPs-legumain and A.C.NPs-legumain are presented in Fig. 2D. The band at 1582 cm^−1^ assigned to the stretching vibration of un-deacetylated amido bond of chitosan, which shifted to 1579 cm^−1^ in the alginic acid coated group. In addition, the 1619 cm^−1^ peak is assigned to the -NH-CO- I bond produced by chitosan -NH_2_ and of alginic acid -COOH groups.

### Characteristics of A.C.NPs-legumain and C.NPs-legumain at Different pH Levels

**Figure 3:**
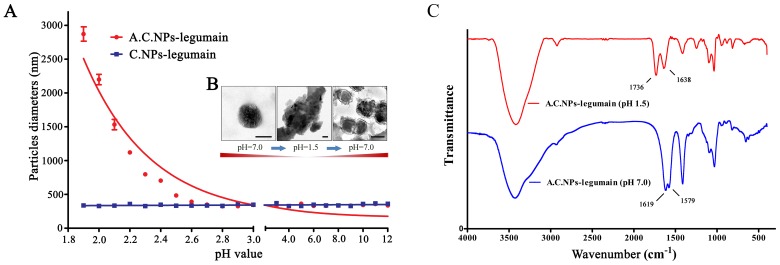
Characteristics of C.NPs-legumain and A.C.NPs- legumain in an acidic environment. (A) Nanoparticles were treated in different acidity levels (pH 1.8∼12) for 2 hours. C.NPs-legumain and A.C.NPs-legumain particle diameter and zeta potential measurements at 37°C. (B) Representative images of A.C.NPs at pH 1.5 (left, scale bar = 1µm) and pH 7.0 (right, scale bar = 100 nm). (C) FTIR spectra of A.C.NPs-legumain at pH 1.5 and pH 7.0.

To evaluate the effect of pH on the physical characteristics of the A.C.NPs and non-coated C.NPs, we measured the size, morphology and FTIR spectra of the nanoparticles at various pH. As shown in Fig. 3A, at pH 1.9, A.C.NPs size was 2.80±0.19 µm measured by size and zeta potential analyzer at 37°C. Particle size decreased rapidly with increasing pH at pH 1.9–2.7. At pH greater than 2.7, there was no size difference between the A.C.NPs and C.NPs. Non-coated C.NPs did not exhibit any significant change in size at different pH levels. Different A.C.NPs forms were observed at different pH levels via TEM (Fig. 3B). At pH 1.5, A.C.NPs aggregated into micrometer-scale particles, but disaggregated into single round particles at pH 7.0. The FTIR spectra of A.C.NPs-legumain at pH 1.5 or 7 are presented in Fig. 3C. The band at 1736 cm^−1^(pH 1.5) is assigned to -COOH of alginic acid because of hydrolysis of -NH-CO- I bond.

### A.C.NPs Protect DNA against Degradation in Simulated Gastric Fluid

**Figure 4:**
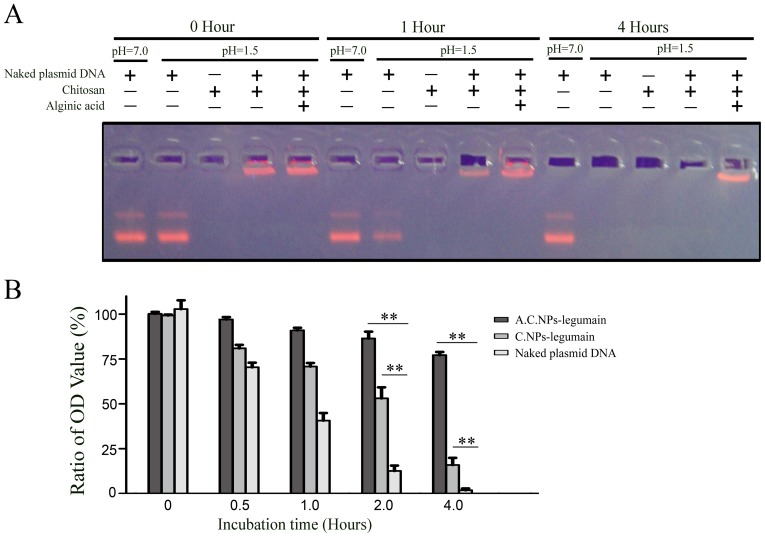
A.C.NPs protect DNA against degradation at low pH. Naked, full-length legumain DNA plasmids, C.NPs-legumain, and A.C.NPs-legumain were each incubated with artificial gastric fluid (pH 1.5) for 0, 0.5, 1, 2 or 4 hours. Naked plasmid DNA was incubated in solution of pH 7.0 for the same time points to serve as a positive control. (A) A representative image of the agarose gel electrophoresis. A lane is observed in the A.C.NPs even after incubation at pH 1.5 for 4 h. (B) Graphical representation of relative OD values. Data are presented as mean ± SD of three independent experiments (**P<0.01).

We further explored the effect of alginic acid modification on acid-resistance since A.C.NPs aggregate at relatively low pH levels. Agarose gel electrophoresis shows that after either 1, 2 or 4 h incubation in simulated gastric fluid (pH 1.5), the degradation of plasmid DNA loaded in the A.C.NPs was significantly less than naked plasmid DNA or plasmid DNA loaded in C.NPs (Fig. 4A, B). Even after 4 h of incubation, more than 78% plasmid DNA remained in A.C.NPs-legumain, while less than 19% and 2% remained in C.NPs-legumain and naked DNA plasmid group, respectively. These data suggested that A.C.NPs can effectively protect DNA against degradation in low pH conditions.

### A.C.NPs Increase DNA Uptake and Expression by Macrophages and Dendritic Cells in the Intestinal Peyer’s Patches

**Figure 5:**
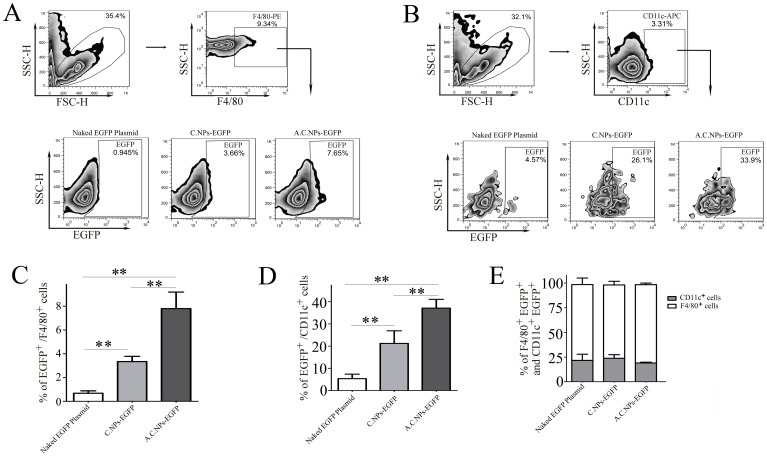
A.C.NPs loaded with DNA pass through the acidic gastric barrier and are taken up by macrophages and dendritic cells in the intestinal Peyer’s patches. Naked EGFP DNA plasmids, C.NPs-EGFP, and A.C.NPs-EGFP were separately given to BALB/c mice (n = 5) via intragastric gavage at a daily dose equivalent to 30 µg plasmid DNA per mouse for three consecutive days. Peyer’s patches were isolated and analyzed by flow cytometry. PE-conjugated F4/80 and APC-conjugated CD11c antibodies were used to stain (A) the macrophages and (B) dendritic cells, respectively. Histograms of the percentage of EGFP-positive (C) macrophages and (D) dendritic cells. (E) Histograms of the ratio of F4/80- or CD11c- positive cells to total EGFP-positive cells. Data are presented as mean ± SD of three independent experiments (**P<0.01; n = 5).

*In vivo* uptake and expression by the macrophages and/or dendritic cells in the intestinal Peyer’s patches is a critical step for oral DNA vaccination. To visualize the uptake and expression of DNA carried by A.C.NPs or C.NPs into the Peyer’s patches, we loaded the nanoparticles with EGFP DNA plasmid. Immunofluorescence staining showed co-staining in EGFP (green) and F4/80 (red) cells (Fig. S1). Flow cytometry results showed that the percentage of EGFP^+^F4/80^+^ (Fig. 5A, C) and EGFP^+^CD11c^+^ (Fig. 5B, D) cells loaded with A.C.NPs were higher than either naked EGFP plasmid or those loaded with C.NPs. The percentage of EGFP^+^F4/80^+^ plus EGFP^+^CD11c^+^ amounted to 100% of EGFP positive cells in the three groups (Fig. 5E). This implies that macrophages or dendritic cells in the Peyer’s patches are the major sources of EGFP expression after oral DNA vaccination. Our data suggest that compared with C.NPs, A.C.NPs increase loaded DNA uptake and expression by macrophages and dendritic cells.

### Effect of A.C.NPs Loaded with Legumain DNA on Tumor Growth

**Figure 6:**
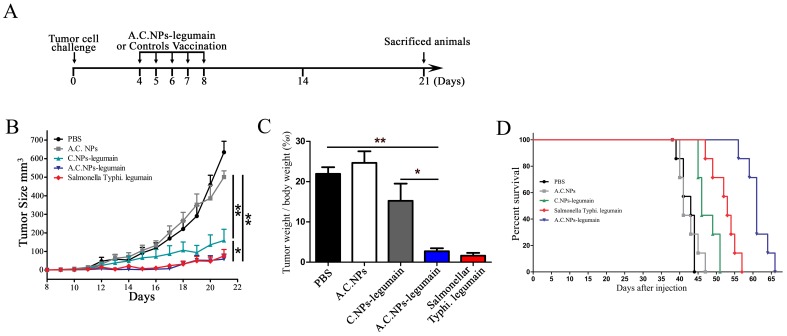
Oral vaccination using A.C.NPs-legumain inhibits tumor growth and increases survival in orthotopic 4T1 breast cancer model. (A) Illustration of the experimental design. (B) Tumor growth curves and (C) ratio of tumor weight vs. body weight in different groups. Data are presented as mean ± SD (*P<0.05, **P<0.01). (D) 50 female BALB/c mice were randomly divided into 5 groups (n = 10). Tumor challenge and oral vaccination were performed as described. A Kaplan-meier survival curve is shown.

To further evaluate the protective effect of oral vaccination with A.C.NPs-legumain, we tested it in the mouse model of orthotopic 4T1 breast cancer. After being challenged with 5×10^4^ 4T1 tumor cells, the mice were orally vaccinated with the same amount of legumain DNA plasmid loaded in different delivery carriers or with the PBS control (Fig. 6A). The tumor size and tumor weight/body weight ratio was significantly less in mice treated with A.C.NPs-legumain versus the PBS control, C.NPs, and empty A.C.NPs group (tumor size: 78.2±31.6 vs 643.6±136.7, 179.3±73.5, 509.1±30.7 mm^3^, respectively; tumor weight/body weight ratio: 4.3±0.7 vs 22.5±2.6, 15.4±7.2, 25.3±6.4%, respectively). There was no significant difference between the A.C.NPs-legumain and legumain DNA vaccine carried with *S. typhi* (Fig. 6B, C).

The survival rate of tumor-bearing mice was significantly higher in the A.C.NPs-legumain group compared with C.NP-legumain group, empty A.C.NPs, legumain DNA vaccine carried with *S. typhi* and PBS (Fig. 6D). These results suggest that A.C.NPs improve the efficiency of oral legumain DNA vaccine against breast cancer in mice.

### Oral Vaccination with A.C.NPs-legumain Improves Immunological Reaction of T Cells Targeting Legumain

**Figure 7:**
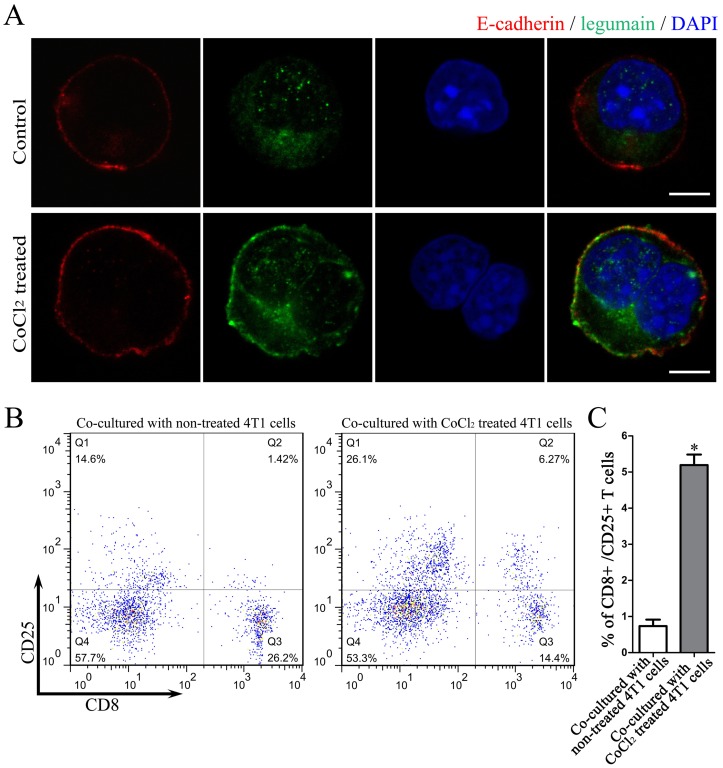
Oral vaccination via A.C.NPs-legumain induces autoimmune CTLs that target legumain. Activated CTLs were accessed by flow cytometry. (A) Representative confocal microscopy images of immnofluorescence staining of 4T1 cells treated with or without CoCl_2_ for 6 h. E-cadherin was used as a cell membrane marker. Significant translocation expression of legumain to the membrane after CoCl_2_ stimulation was observed. (B) Representative flow cytometry histogram of splenocytes co-cultured with 4T1 cells treated with or without CoCl_2_. (C) Histogram of the percentages of CD8 and CD25 double positive cells. Data are presented as mean ± SD of five independent experiments (*P<0.01).

**Figure 8:**
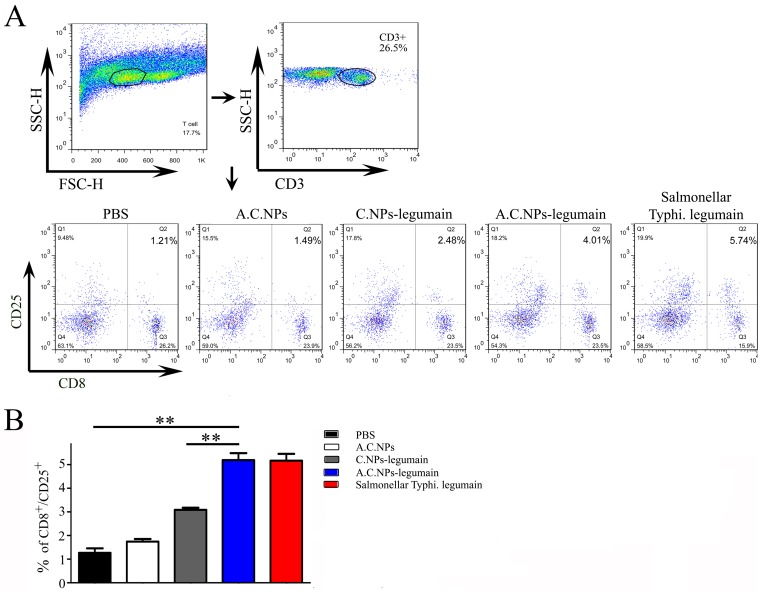
Oral vaccination via A.C.NPs-legumain enhances the autoimmune response of CTLs compared with other treatment. Animals (n = 5) were grouped and treated as described. Upon sacrifice, splenocytes were isolated and co-cultured with 4T1 cells, which had been pretreated with CoCl_2_ for 24 h. The percentage of CTLs activity was measured via flow cytometry. (A) Representative histograms of different groups. (B) The percentages of CD8 and CD25 double positive cells. Data are presented as mean ± SD (**P<0.01).

We further explored the mechanism underlying the protective effect of A.C.NPs-legumain against breast cancer. It has been reported that legumain translocates to the plasma membrane under hypoxic stimulation [29]. Hypoxia is common in tumor environment due to the rapid proliferation of tumor cells and an insufficient blood supply [30], [31]. Immunofluorescence staining showed evidence of translocation of legumain expression in cultured 4T1 cells after CoCl_2_ treatment (Fig. 7A). Splenocytes isolated from A.C.NPs-legumain treated mice were co-cultured with CoCl_2_ treated or non-treated 4T1 cells. Flow cytometry results indicate that the ratio of CD8^+^CD25^+^ T cell increased significantly after co-culture with CoCl_2_ treated 4T1 cells (Fig. 7B, C). Splenocytes isolated from mice treated with A.C.NPs-legumain co-cultured with 4T1 cells treated with CoCl_2_ exhibited a 1.66-, 2.75- and 3.91- fold higher number of activated T cells than when treated with C.NPs-legumain, empty A.C.NPs or PBS, respectively. Treatment with A.C.NPs-legumain group and legumain DNA with *S. typhi.* carrier group showed similar results. (Fig. 8A, B).

An interesting additional finding was that the percentage of active regulatory CD4^+^CD25^+^ cells following A.C.NPs-legumain and empty A.C.NPs treatment is significantly lower than that for the other three groups (Fig. S2A, B). This implies that suppression of T cells immunity by A.C.NPs might contribute to the protective effect of oral legumain DNA vaccine against tumors.

## Discussion

**Figure 9:**
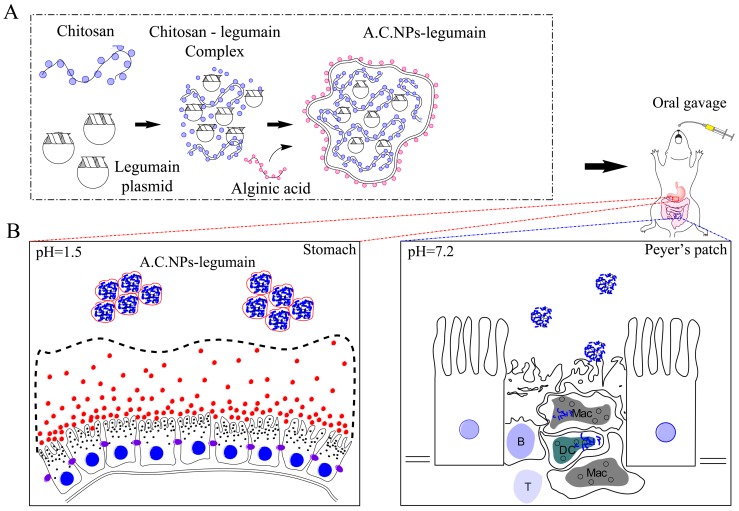
Diagram shows the protective effect of A.C.NPs on DNA against enzymatic and acidic degradation. (A) Schematic of A.C.NPs-legumain preparation. (B) Schematic representation of A.C.NPs-legumain passing through the acidic gastrointestinal track and taken up by antigen-presenting cells in the intestinal Peyer’s patches.

Development of non-toxic and efficient delivery systems has always been a challenge with the application of DNA vaccines [32], [33]. As a result, the biocompatible and biodegradable features of chitosan nanoparticles have gained more attention as a carrier for oral DNA vaccination [34]. Although many efforts have been put into the design and modification of chitosan nanoparticles, a viable oral delivery system for DNA vaccines is still lacking [33]. One major obstacle to achieving satisfactory oral vaccinations in the DNA degradation results when exposed to the acidic gastric environment and the subsequent decreased uptake via lymphoid tissue in the gastrointestinal tract [35], [36], [37]. We demonstrated here, that compared with plain chitosan nanoparticles, A.C.NPs protect DNA against degradation in stimulated gastric fluid (pH 1.5). At pH 7.0, alginic acid forms amide bonds with DNA-loaded chitosan nanoparticles by reaction of alginic acid carboxyl groups with chitosan amino groups. Interestingly, after modification with alginic acid, DNA-loaded chitosan nanoparticles demonstrate a distinct trend as pH values increase. A.C.NPs tend to aggregate into micrometer-scale complexes at pH 1.5, whereas they disperse into smaller particles as pH increases. This difference might be attributed to the hydrolysis of the amide bond in the acidic condition and the subsequent decomposition of the cross-linked structure between the carboxyl groups of alginic acid and amino groups of chitosan. Excess alginic acid is insoluble at pH 1.5 and potentially forms an amorphous shell on the nanoparticle surface; protecting DNA against enzymatic and acidic degradation (Fig. 9A). Uptake and expression by antigen-presenting cells in Peyer’s patches is a critical step toward successful oral DNA vaccination [18], [20]. We visualized the expression of DNA plasmids when loaded in diverse delivery carriers using enhanced green fluorescence protein DNA plasmids. Our results suggest that after leaving the acidic gastric environment and entering the intestines, DNA loaded in A.C.NPs are effectively taken up and expressed by the Peyer’s patches. Expression of EGFP was predominately located in macrophages and dendritic cells. It appears as though modification with alginic acid largely increased the amount of EGFP expression in Peyer’s patches after oral administration (illustrated schematically, Fig. 9B). The absorption promoting effect of C.NPs has been extensively studied. Chitosan has been shown to exhibit increased mucoadhesion due to electrostatic interaction, as well as influence the transient opening of tight junctions; both of which may promote the transportation of a loaded DNA vaccine across the mucosal barrier [38]. In addition, studies also indicated that chitosan uptake increases the accumulation and activation of macrophages, providing an extra advantage in improving vaccination efficiency.

A noninvasive, controllable, stable, and effective DNA vaccine for cancer therapy would offer a new therapeutic approach. We utilize a full-length legumain DNA vaccine and the murine orthotopic 4T1 breast cancer model to evaluate effectiveness as an oral carrier. Our data demonstrate that legumain DNA vaccine carried with A.C.NPs exhibits a similar, if not better, effect on suppressing tumor growth and prolonging survival of tumor-burdened animals compared with both attenuated *S. typhi*-based vaccine and vaccine carried by C.NPs. Luo et al. have already reported that legumain is specifically expressed by tumor-associated macrophages (TAMs) in the tumor microenvironment [6]. Autoimmunity established via vaccination with a legumain DNA vaccine destroys TAMs and remodels the immunosuppressive milieu that benefits tumor development. Recent studies reveal that legumain overexpression is detected in the tumor cells themselves in some tumor models. Given this, it may be the autoimmunity targeting legumain would have a dual effect. It may help remodel the microenviroment that supports tumor survival; while at the same time act to deplete tumor cell populations directly. Interestingly, oral vaccination with legumain DNA increases the amount of activated CTLs (CD8^+^CD25^+^) targeting legumain. Moreover, particles with alginic acid inhibit the activated regulatory T cells (CD4^+^CD25^+^) aimed at legumain (Fig. S2). This might explain the longer survival time of mice vaccinated with A.C.NPs compared with those receiving a *S. typhi*-based vaccine.

In summary, we utilized A.C.NPs as a delivery carrier for an oral DNA vaccine and evaluated their morphological and physical characteristics. We identified their degradation properties in simulated gastric conditions, as well as the protective effect on prolonging survival time and tumor growth suppression in the orthotopic breast cancer model. Although numerous issues need to be addressed before practical application of oral DNA vaccine can occur, our study may provide insight into a potential therapeutic strategy for breast cancer treatment and has moved a step toward its potential clinical application.

## Supporting Information

Figure S1 Oral administrated A.C.NPs are taken up by F4/80 positive cells in Peyer’s patches. Naked EGFP DNA plasmid, C.NPs-EGFP and A.C.NPs-EGFP were separately given to BALB/c mice via intragastric administration at a daily dose of 30 µg plasmid DNA per mouse for three consecutive days. Peyer’s patches in small intestines were fixed and prepared into 5-µm-thick slides. Antibody of F4/80 was used to perform the immunofluorescence staining. Representative images indicated that, in Peyer’s patches, the scope of EGFP expression (green) was significantly stronger in A.C.NPs-EGFP group comparing with naked EGFP DNA plasmid or C.NPs-EGFP group. Moreover, overlay of EGFP (green) and F4/80 positive cells (red) is detected in mice treated with A.C.NPs-EGFP. Scale bar = 50 µm.
(TIF)

Figure S2 Oral vaccination via A.C.NPs-legumain significantly inhibited regulatory T cells. Animals were grouped and treated as described. Upon sacrifice, splenocytes were isolated (n = 5) and co-cultured with 4T1 cells pretreated with CoCl_2_ for 24 h. The percentage of regulatory T cell was measured via flow cytometry. (A) Histogram of flow cytometry results. (B) Graphical representation of the percentage of CD4 and CD25 double positive cells. Data are presented as mean ± SD (*P<0.05).
(TIF)
