# Supplementary figures and images for: Alginic Acid-Coated Chitosan Nanoparticles Loaded with Legumain DNA Vaccine: Effect against Breast Cancer in Mice
Source: PLoS One. 2013 Apr 5;8(4):e60190. doi: 10.1371/journal.pone.0060190 (PMC3618226; doi:10.1371/journal.pone.0060190)

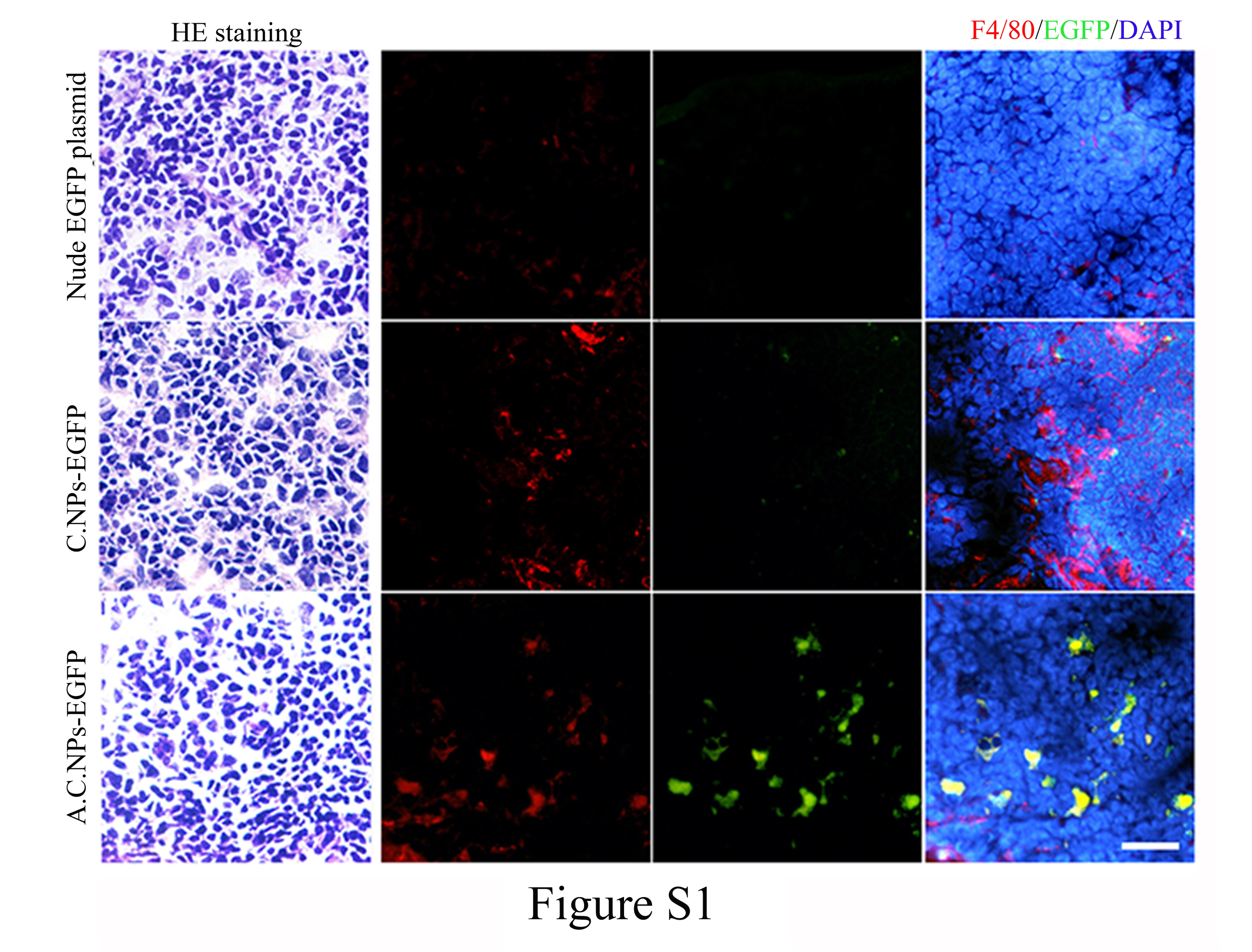

Supplement: Figure S1 — Oral administrated A.C.NPs are taken up by F4/80 positive cells in Peyer’s patches. Naked EGFP DNA plasmid, C.NPs-EGFP and A.C.NPs-EGFP were separately given to BALB/c mice via intragastric administration at a daily dose of 30 µg plasmid DNA per mouse for three consecutive days. Peyer’s patches in small intestines were fixed and prepared into 5-µm-thick slides. Antibody of F4/80 was used to perform the immunofluorescence staining. Representative images indicated that, in Peyer’s patches, the scope of EGFP expression (green) was significantly stronger in A.C.NPs-EGFP group comparing with naked EGFP DNA plasmid or C.NPs-EGFP group. Moreover, overlay of EGFP (green) and F4/80 positive cells (red) is detected in mice treated with A.C.NPs-EGFP. Scale bar = 50 µm. (TIF) [file pone.0060190.s001.tif]

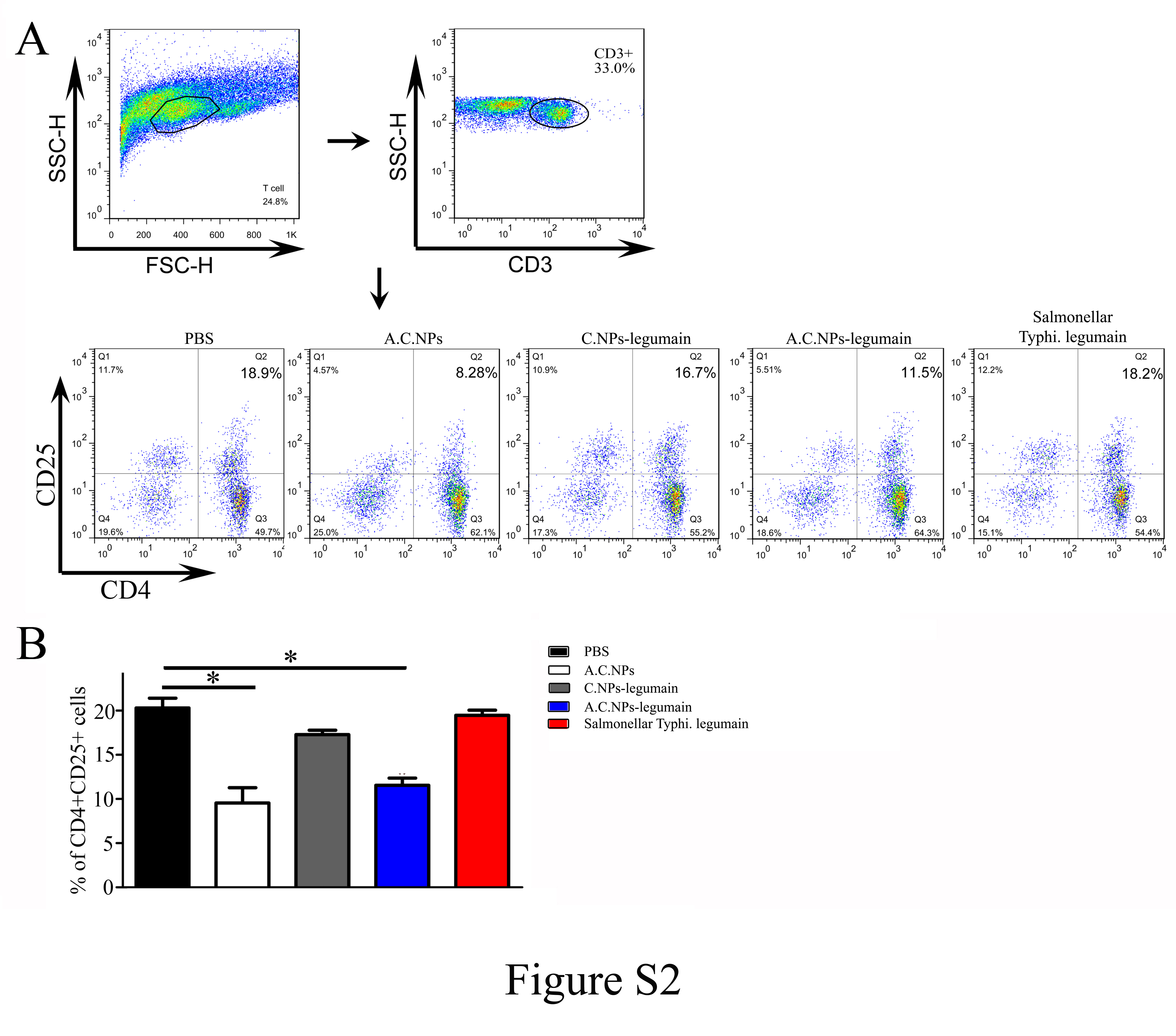

Supplement: Figure S2 — Oral vaccination via A.C.NPs-legumain significantly inhibited regulatory T cells. Animals were grouped and treated as described. Upon sacrifice, splenocytes were isolated (n = 5) and co-cultured with 4T1 cells pretreated with CoCl2 for 24 h. The percentage of regulatory T cell was measured via flow cytometry. (A) Histogram of flow cytometry results. (B) Graphical representation of the percentage of CD4 and CD25 double positive cells. Data are presented as mean ± SD (*P<0.05). (TIF) [file pone.0060190.s002.tif]
